# Analysis of disease profile, and medical burden by lead exposure from hospital information systems in China

**DOI:** 10.1186/s12889-019-7515-5

**Published:** 2019-08-27

**Authors:** Han Song, Jianchao Liu, Zipeng Cao, Wenjing Luo, Jing-Yuan Chen

**Affiliations:** 1 Department of Health Service, PLA General Hospital, Beijing, 100853 China; 2 Department of Occupational and Environmental Health, and the Ministry-of-Education’s Key Laboratory of Hazard Assessment and Control in Special Operational Environment, School of Public Health, Air Force Medical University, No.169, Changlexi Road, Xi’an, 710032 China

**Keywords:** Pb, Disease profile, Medical burden, Medical big data

## Abstract

**Background:**

Though lead (Pb)-gasoline has been banned for decades in China, Pb continues to be a vital risk factor for various diseases. Traditional studies, without large sample size, were unable to identify explicitly the associations among Pb, its disease profile, and the related medical burden. This study was designed to investigate: 1) current status of blood Pb levels; 2) Pb-associated disease profile, medical burden, as well as impact factors.

**Methods:**

Research subjects were patients who visited military hospitals and were required to test their blood Pb levels by doctors between 2013 and 2017. The large sample size and area coverage may, to a large extent, reveal the characteristics of Pb exposure in the whole Chinese population. Information of patients’ electronic medical records was extracted using Structured Query Language (SQL) in Oracle database. The spatial, temporal, and population distribution of their blood Pb levels were tested, to illustrate the association of Pb exposure with diseases’ profile, and medical burden. Non-parametric tests were applied to compare the differences of Pb levels among various groups.

**Results:**

The blood Pb concentration showed a positively skewed distribution by Kolmogorov-Smirnov test (D = 0.147, *p* < 0.01). The blood Pb concentration of Chinese patients was 28.36 μg/L, with the lowest blood Pb levels, 4.71 μg/L, found in patients from Guangxi Zhuang Autonomous Region, and the highest, 50 μg/L, in Yunnan province. Han Chinese patients’ Pb levels were significantly lower than other minorities groups (z-score = − 38.54, *p* < 0.01). Average medical cost for Pb poisoning was about 6888 CNY for Chinese patients. Pb levels of patients with malignant neoplasm of lung, 45.34 μg/L, were far higher than malignant neoplasm of other respiratory, and intrathoracic organs, 24.00 μg/L (z-score = − 2.79, *p* < 0.01).

**Conclusions:**

This study reported current status of blood Pb levels for patients who once visited military hospitals, partially representing the whole Chinese population. The result shows that Pb poisoning is still imposing marked economic burdens on patients under Pb exposure. Association of Pb with lung cancer may open up new areas for Pb-induced toxicology. The research strategy may advance toxicological studies in the aspect of medical data mining.

## Background

Lead (Pb)-gasoline has been phased out in China since the 1980s, and was completely banned all over China since 2000 [1]. Though laws were enforced rigorously to remove the use of Pb sources in commercial, and industrial sectors, in China, Pb exposure continues to be an important risk factor, affecting almost every organ, and system in the human body [2]. The current mass epidemiological investigations mainly focused on occupational Pb poisoning, or childhood Pb exposure [3, 4]. In recent years, medical big data has made it possible to identify the association of Pb exposure with disease profile based on the massive/large sample size of cross-sectional studies.

Pb is generally known as a widespread environmental pollutant, and causes a wide range of toxic effects, with no manifest biological roles. Traditional epidemiological studies, by using cross-sectional, case-control, cohort, or case report methods, have elucidated the relations between Pb, and certain kinds of diseases. Their research findings pointed to the association between Pb exposure and circulatory system diseases, e.g., hypertension, intracerebral hemorrhage, atherosclerosis and acute ischemic stroke [5–7], and a great affinity of the cerebral endothelial cells to Pb suggested that Pb concentrations in endothelial cells were much higher than that in other brain cell types [8]. Previous studies also uncovered that vasogenic edema, increased Blood-Brain Barrier (BBB) permeability as well as cerebellar hemorrhage were significant manifestations of Pb-induced damage of microvessels in developing brain [9, 10]. Nephrotoxicity may occur as a result of Pb exposure because kidney was the main excretion route, only by which intracorporal Pb would be eliminated [11]. Chronic Pb exposure at levels above 700–800 μg/L was an established indicator for chronic kidney disease (CKD) [12]. Proximal tubular cells of the renal tubules accumulated Pb through specific Pb-binding proteins as the form of typical intracellular inclusions [13, 14].

The research subjects of our study are outpatients and inpatients who were required to test blood Pb concentrations in military hospitals for various symptoms. International Statistical Classification of Diseases, and Related Health Problems (ICD-10) code was implemented by coders of Medical Records Management Division based on doctor’s clinical diagnosis [15]. Thus, with this convenience brought by ICD in exchanging and sharing disease information across various hospitals, our study aimed to investigate (1) current status of blood Pb levels for patients who visited military hospitals; (2) the association of Pb exposure with the whole diseases’ profile, and corresponding medical burden; (3) associations between covariates and blood Pb levels. The results from this study may help us understand the underlying Pb-induced toxicity in human body system.

## Methods

### Study population

Study population comprises patients receiving whole blood Pb concentrations test in treatments. The present study was approved by the institutional review boards of Air Force Medical University (Registry no. KY20173346–1), and all studies were conducted in accordance with the ethical principles for medical research involving human subjects as defined in the Declaration of Helsinki. A total number of 294,402 outpatients/inpatients of all military hospitals between 2013 and 2017 were included as subjects by non-probability sampling using SQL expression of Oracle databases. Records lacking in any primary keys, including hospital information, patient’s identity, or visiting times, were excluded. Given that China is a country with 56 ethnic groups, we also used the self-report ethnic information to reveal in vivo Pb heterogeneity levels in different ethnic groups. Pb content measured by Atomic Absorption Spectroscopy (AAS) in whole blood was expressed as micrograms per liter (μg/L).

### Data resource

This study aims to provide a comprehensive review of medical big data collected from military hospitals, which serves not only the military personnel, but also civilians, taking up a significantly high proportion of Chinese health services. The data was collected in the treatment, which includes patients’ demographic information, vital signs, diagnoses of diseases, medical orders, examination reports, lab tests results, drugs, medical supplies or even infections. Patients’ information was recorded in the processes of registration, ward management, medical order, auxiliary examination, and even in surgery. Besides, the information flow within departments of each hospital were documented by Admission-Discharge-Transfer (ADT), document entry, drug placement, or medical record cataloging. Medical costs were composed of bed, clinical use of drug, laboratory examination, X-ray, treatment, blood test, operation, nursing care, etc.

### Data mining

Data processing is done by Structured Query Language (SQL) in Oracle database 10 g. Each medical record consists of information including patient’s demographic characteristics, vital signs, diagnoses, ADT, surgeries, medical orders, examination reports, lab tests, drugs, medical supplies, medical costs, nursing care, infections, and treatment outcomes. First, we extracted inpatients from this data set, those who had received blood Pb concentration test in hospitals. The primary key of each patient’s record includes hospital code, patient ID, and visit times. Medical costs as well as diagnosis information were included during the analytical process. With a reference to the above indicators, the main diagnostic results, and other important information were retrieved from this relational database for further correlation analysis of Pb exposure.

### Study setting

Data sets from more than 200 military hospitals between 2013 and 2017 were obtained, with each hospital equipped with a Hospital Information System (HIS) for uniformed services as well as similar data dictionary. People’s Republic of China (PRC) covers an area of approximately 9,600,000 km^2^, governing 23 provinces, five autonomous regions, four direct-controlled municipalities (Beijing, Tianjin, Shanghai, and Chongqing), and the special administrative regions of Hong Kong, and Macau. The hospitals in this study are mostly located in the capital cities of these provinces. Patients were labeled with two geographic tags, their birthplaces implicated in their ID card, and the workplace implicated in the postal code of China. We intended to outline Pb spatial distribution in patients by these two geographic tags.

### Statistical analysis

Our analysis is done on the basis of patients’ disease profile, and medical burden of Pb exposure in more than 100 cities, which are either provincial capitals or cities in key regions, accounting for about 30% of the major cities in China. Personal profiles were compiled based on demographic factors, including gender, age, or blood types of patients receiving blood Pb test. We counted Pb values every single time when patients got Pb tested for multiple times. Kolmogorov-Smirnov test was employed to identify the distribution of whole blood Pb concentration, with Q-Q plot applied to visualize the results. To analyze our data by a cross-sectional approach, we evaluated the differences between two independent samples by Wilcoxon rank sum test, or multiple independent samples by Kruskal-Wallis H test. Medical cost structure was analyzed based on all details of medical expenses. Data were analyzed, or illustrated using GraphPad Prism 6, and Python (version 5.1.0; Anaconda3). Values were considered statistically significant at *p* < 0.05.

## Results

### Demographic characteristics of patients receiving whole blood Pb test

**Fig. 1:**
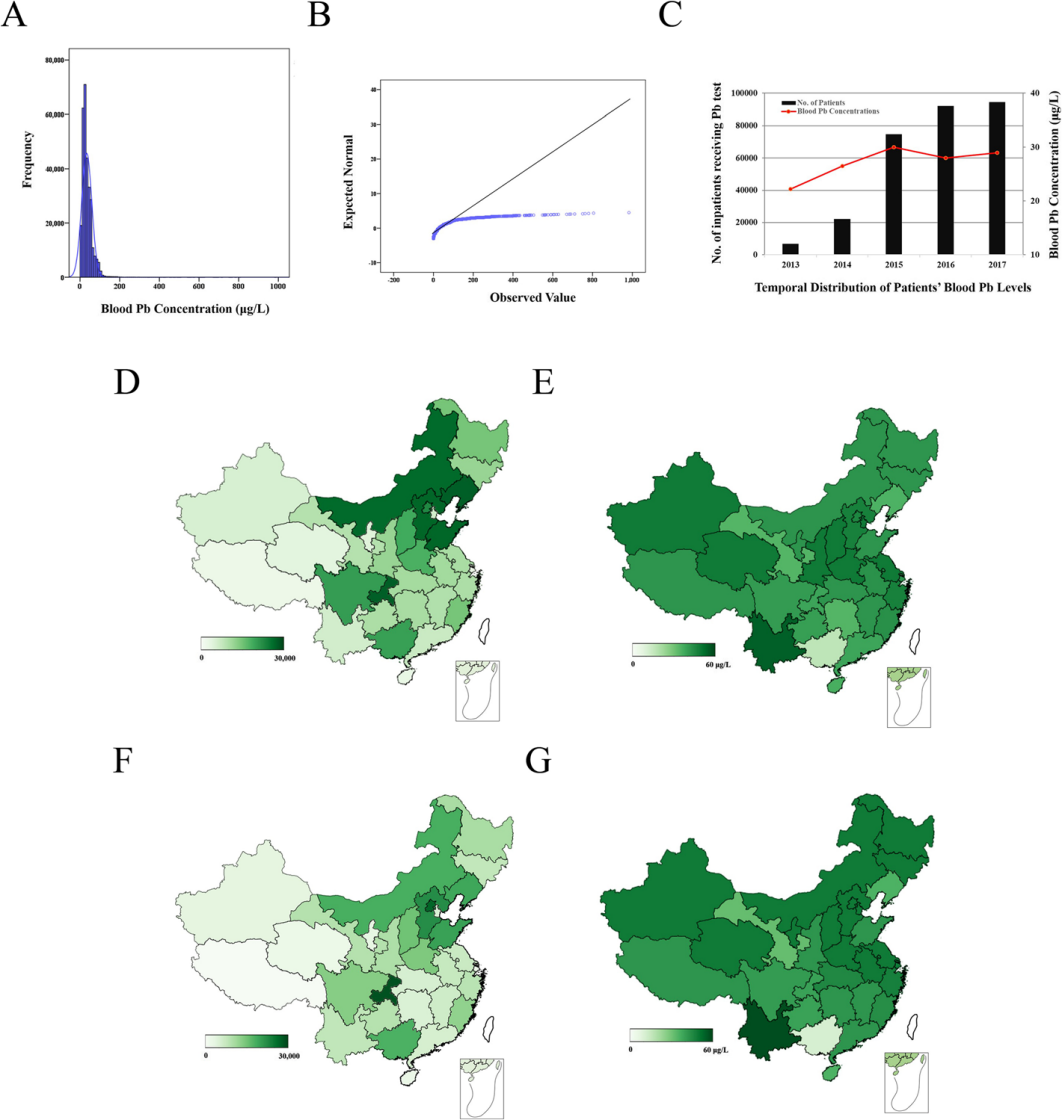
Temporal, and spatial distribution of patients receiving whole blood lead (Pb) test. **a** Blood Pb frequency distribution among all the patients. **b** Patients’ Pb distribution test with Q-Q plot. **c** Amount of data as well as blood Pb concentrations from 2013 to 2017. The red lines indicate the median of blood Pb levels at each year. Bar chart depict the sample size converting from 2013. **c** and **d** Sample size (**c**), and blood Pb concentrations (**d**) indicated with heating map of patients receiving Pb test born in each region in China. **e** and **f** Sample size (**e**), and blood Pb concentrations (**f**) of patients receiving Pb test worked in each region in China. Color corresponds to number of patients (**c**, **e**), or Pb levels expressed with μg/L (**d**, **f**)

To choose appropriate statistics for Pb, we tested the distribution of whole blood Pb concentrations among 294,402 patients who received heavy metal blood test. The blood Pb concentration showed a positively skewed distribution with a mean of 33.71 μg/L, yet a median of 28.36 μg/L (Fig. 1-a, b).

### Temporal and spatial distribution

The big data sets were starting to build in 2013. At that time, data size of Pb tests was only 6802. After that, the data size increased progressively to 92,159 and 94,449 patients in 2016, and 2017, respectively. Along with this data expansion, the median of blood Pb concentration in patients rose from 22.2 μg/L in 2013 to 29.0 μg/L in 2017 (Fig. 1-c).

Patients born in Hebei (23,941), Shandong (23,916), Inner Mongolia (23,190), Beijing (17,052), but worked in Chongqing (27,347), Beijing (23,901), Hebei (16,416), Shandong (13,136), Liaoning (12,261), Inner Mongolia (11,133), were required to test their Pb exposure levels (Fig. 1-d, f). Interestingly, blood Pb concentrations of patients born in Guangxi were the lowest, 4.71 μg/L, while that of Yunnan province was the highest, 50 μg/L (Fig. 1-e). Similar to the results of their birthplace, blood Pb concentrations of patients worked in Guangxi were also the lowest, 2.05 μg/L, and patients in Yunnan province was the highest, 56 μg/L (Fig. 1-g).

### Blood Pb levels in various population structures

**Fig. 2:**
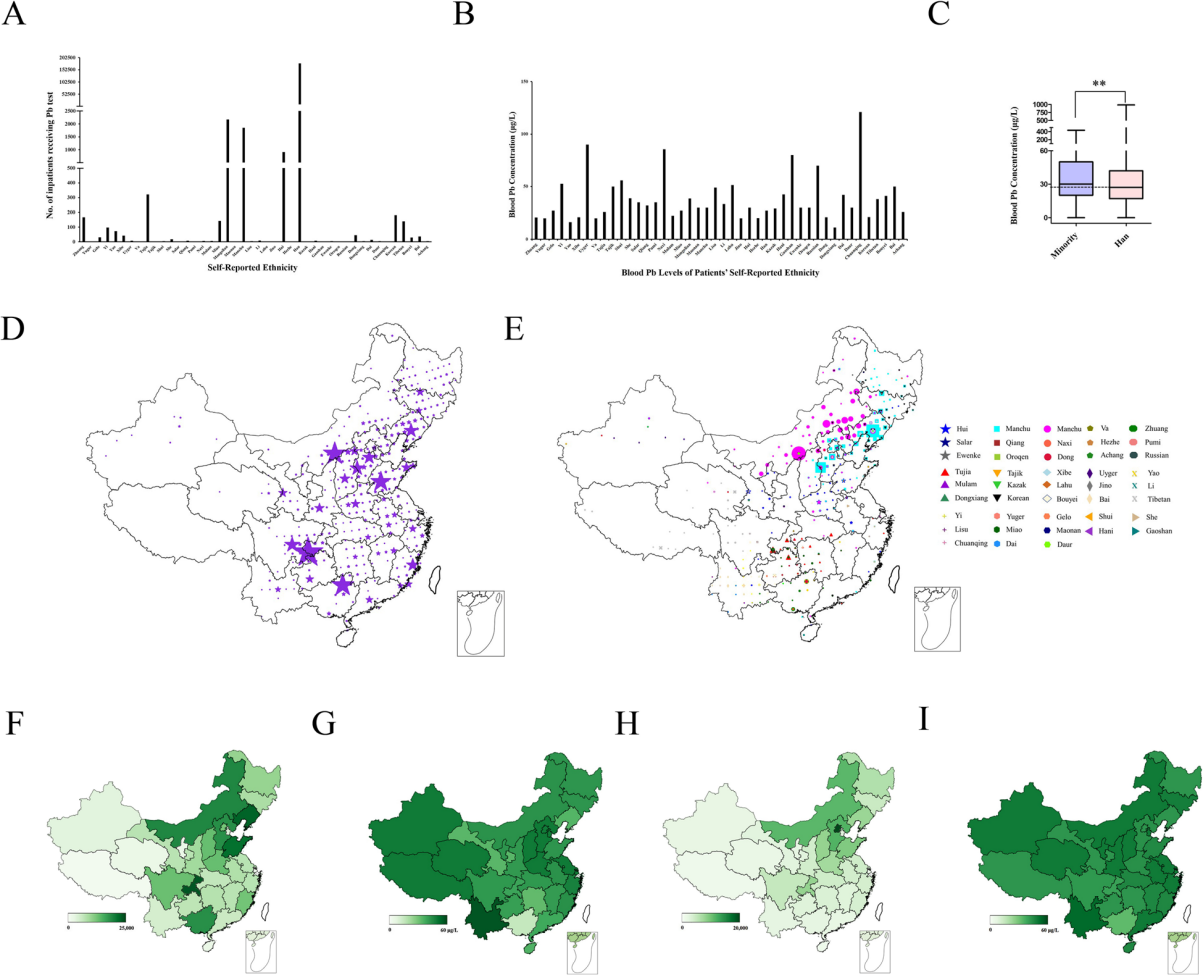
Blood Pb levels from various population structures. **a** and **b** Number of patients (**a**), or blood Pb concentrations (**b**) within various ethnic groups reported by patients themselves. **c** Box plot of Pb levels between Han, and other 43 ethnic minorities. Asterisks depict a statistically significant difference (*p* < 0.01) with Wilcoxon rank sum test. **d** and **e** Detailed geographical distribution of the Han Chinese (**d**), and other 43 minorities (**e**). Size of the symbols reflect the number of individuals. **f** and **g** Sample size (**f**), and blood Pb concentrations (**g**) indicated with heating map of Han Chinese patients receiving Pb test born in each region in China. **h** and **i** Sample size (**h**), and blood Pb concentrations (**i**) of other 43 ethnic minorities. Color corresponds to number of patients (**f**, **h**), or Pb levels (**g**, **i**)

Among all the ethnic groups, Han Chinese has the largest population in China, taking up about 96.56% of the total patients. As for the rest 55 ethnic minorities, Mongolia (2170), Manchu (1848), Hui (914), Tujia (321) accounted for over 80% (Fig. 2-a). Han Chinese, despite their vast proportion in patients, showed a much lower blood Pb levels than some other minorities, 27 μg/L. Pb concentration of Chuanqing, Uyger, Naxi, Gaoshan, Russian patients, were higher than other groups, with a concentration of 121 μg/L, 90 μg/L, 85.5 μg/L, 80 μg/L, 70 μg/L respectively (Fig. 2-b). Median of Han was 27 μg/L, significantly lower than all the other 43 ethnic minorities, 30 μg/L (Fig. 2-c).

Herein, we tried to investigate the geographical characteristics by referring to the birthplace code of Han Chinese, and the minorities. We labeled patients with latitude and longitude according to their birthplace codes in county areas in China. The detailed geographical information of Han patients was shown in Fig. 2d. Meanwhile, Fig. 2e displayed the birthplace information of other 43 minorities by various labels. The majority of Han patients who received blood Pb test came from Chongqing (22,037), Liaoning (20,420), Shandong (18,350), Inner Mongolia (14,514), Hebei (13,382), Beijing (6044) (Fig. 2-f), whereas over half of minorities patients were born in Beijing (19,699), Hebei (6823), Inner Mongolia (6298) (Fig. 2-h). Blood Pb concentrations of Han patients from Guangxi, Hunan, Gansu, Liaoning were 3.9 μg/L, 17.9 μg/L, 19.11 μg/L, 20 μg/L, respectively, which are significantly lower than the other regions, while that of Yunnan province marked the highest, 54 μg/L (Fig. 2-g). Likewise, such geographical characteristics also existed among the ethnic minority groups (Fig. 2-i).

### Personal medical burden derived from Pb exposure

**Fig. 3:**
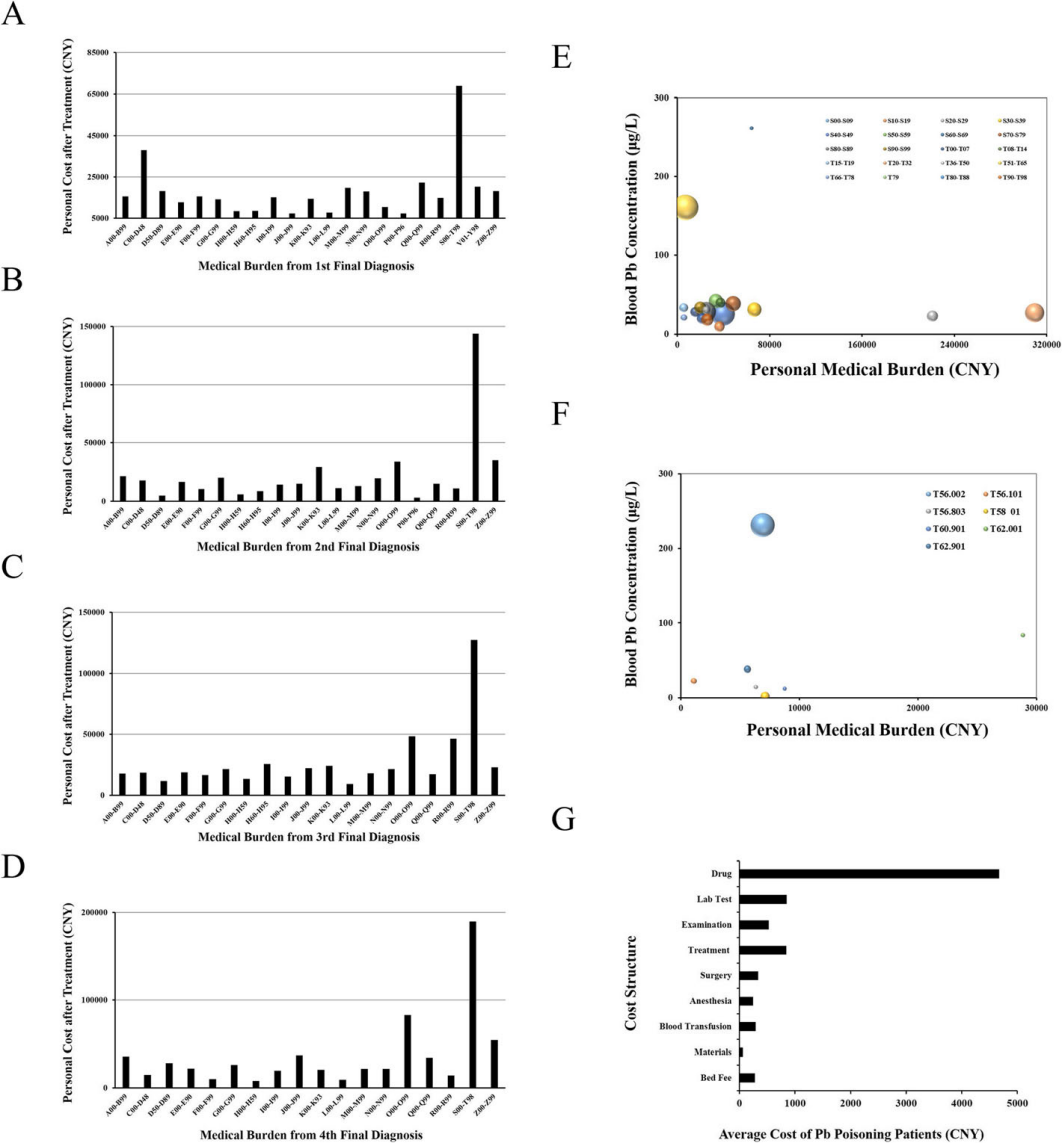
Personal medical burden after treatment derived from Pb exposure. **a**, **b**, **c**, and **d** inpatients’ medical cost within the ICD-10 block for the first (**a**), second (**b**), third (**c**), and fourth (**d**) final diagnosis. **e** Bubble charts of personal medical burden, and blood Pb concentration of subgroups within injury, poisoning, and certain other consequences of external causes (Block S00–T98) for the first final diagnosis. **f** Bubble charts of personal medical burden, and blood Pb concentration of specific items with the subgroups (T51-T65). Size of the symbols reflect the number of individuals

To identify the economic burden driven by Pb exposure on individual patients, we calculated average cost per inpatient of 4 major final diagnoses separated by each ICD-10 block. The medical costs were expressed in Chinese Yuan (CNY). The results showed that the block “inpatients with injury, poisoning, and certain other consequences of external causes (S00-T98)” takes up the highest medical cost compared with all the other blocks from first to fourth final diagnosis (Fig. 3-a, b, c, d). As expected, ICD-10 code for Pb poisoning falls into the subgroup “toxic effects of substances chiefly nonmedicinal as to source (T51-T65)”. Thus, we further investigated the diseases based on ICD-10 classification system within the block S00-T98 in the first final diagnosis. Medical burden of different diseases groups were assessed and compared by bubble charts. Blood Pb concentration of T51-T65 were 160.60 μg/L, higher than the threshold, 100 μg/L, of Pb poisoning (Fig. 3-e). These inpatients’ medical cost may be largely ascribed to their Pb exposure. Further, within the subgroup T51-T65, specific items were utilized to categorize diseases. Average cost for “Pb poisoning (T56.002)” was 6888 CNY (Fig. 3-f). We further analyzed the structure of medical cost afforded by patients with Pb poisoning, where drug cost took the large proportion of the medical expenses, 4670 CNY (Fig. 3-g).

### Association of Pb exposure with diseases occurrence

**Fig. 4:**
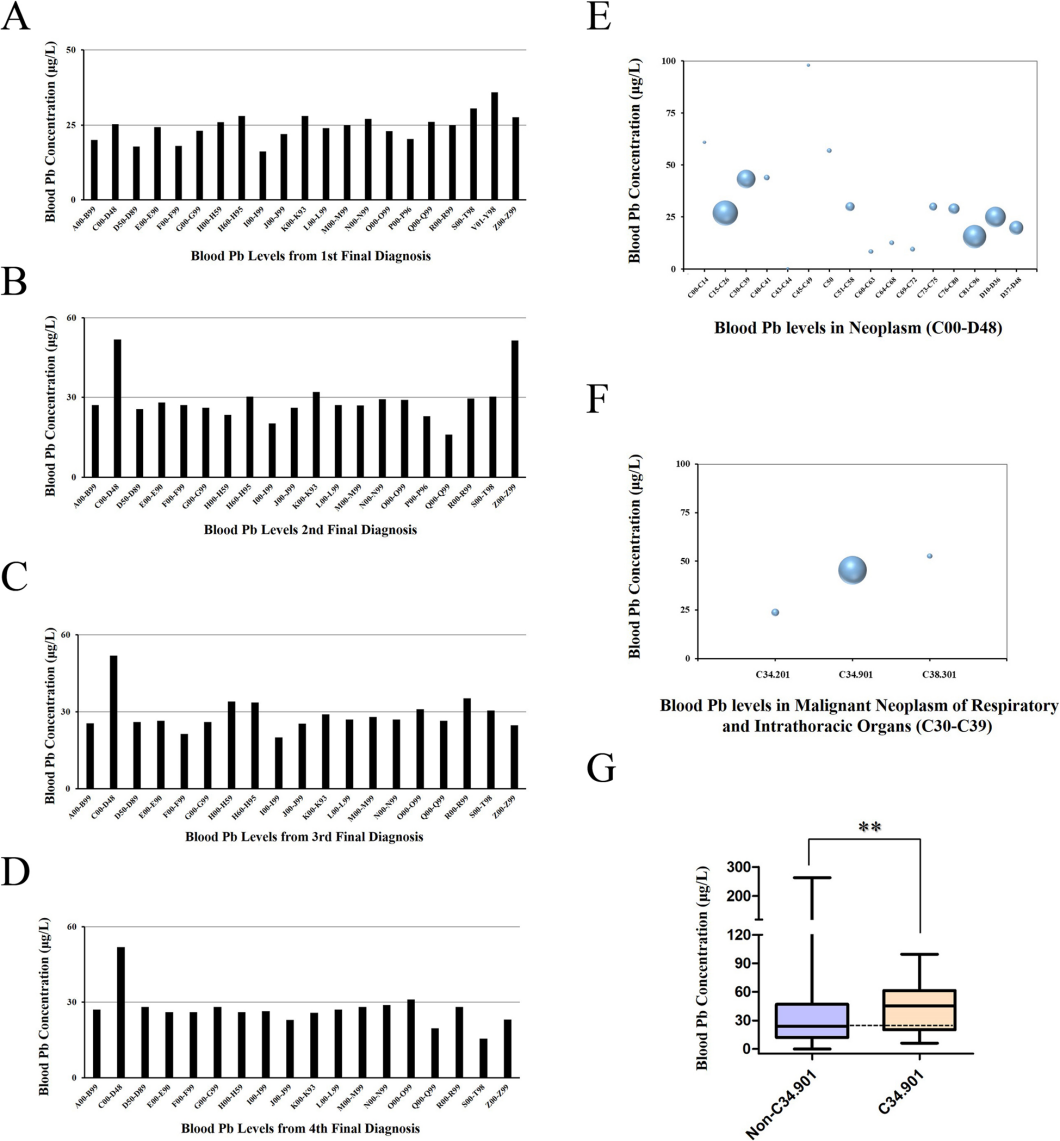
Association of Pb exposure with diseases occurrence. **a**, **b**, **c**, and **d** Blood Pb concentration of inpatients within the ICD-10 block for the first (**a**), second (**b**), third (**c**), and fourth (**d**) final diagnosis. **e** Bubble charts of blood Pb concentration of subgroups within neoplasm (Block C00–D48) for the first final diagnosis. **f** Bubble charts of blood Pb concentration of specific items with the subgroups (C30-C39). Size of the symbols reflect the number of individuals. **g** Box plot of Pb levels between malignant neoplasm of lung (C34.901), and non-C34.901 inpatients within the C00–D48. Asterisks depict a statistically significant difference (*p* < 0.01) with Wilcoxon rank sum test

In an attempt to identify the association between Pb exposure and diseases occurrence, blood Pb concentration were recorded based on the major diagnosis separated by ICD-10 blocks. “Neoplasms (C00-D48)” showed relatively lower blood Pb levels, 25.32 μg/L, in the first final diagnosis (Fig. 4-a), but over 50 μg/L in the second, third, and even fourth final diagnosis, far higher than other blocks (Fig. 4-b, c, d). C00-D48 deals with neoplastic conditions including cancer, carcinoma in situ, and benign tumors. Among the C00-D48 group, diseases mostly included “malignant neoplasms, digestive organs (C15-C26)”, “malignant neoplasms, respiratory system, and intrathoracic organs (C30-C39)”, “malignant neoplasms, stated, or presumed to be primary, of lymphoid, haematopoietic, and related tissue (C81-C96)”, “benign neoplasms (D10-D36)”. However, inpatients with respiratory system and intrathoracic organs disorders (C30-C39) reported the highest Pb concentrations, 43.32 μg/L (Fig. 4-e). In this subgroup C30-C39, 31 inpatients were reported as “malignant neoplasm of lung (C34.901)” under the specific items (Fig. 4-f). Within the block C00-D48, we compared the differences of C34.901, and non-C34.901 inpatients using non-parametric rank test. Blood Pb concentrations of C34.901 inpatients, 45.34 μg/L, were unexpectedly higher than non-C34.901, 24.00 μg/L (z-score = − 2.79, *p* < 0.01) (Fig. 4-g).

Additionally, the block “factors influencing health status, and contact with health services (Z00-Z99)” also reported higher blood Pb levels, 51.38 μg/L in second final diagnosis. “Persons with potential health hazards related to family, and personal history, and certain conditions influencing health status (Z80-Z99)” took the most part. Similar to the association between malignant neoplasm and Pb levels, the median of blood Pb concentration among patients with “personal history of malignant neoplasm (Z85)” was 51.01 μg/L. One patient with “personal history of malignant neoplasm of trachea, bronchus, and lung (Z85.1)” reported 61.53 μg/L blood Pb concentration.

Meanwhile, to further elucidate the relation between Pb exposure and diseases profile, the prevalence rate of inpatients with blood Pb concentrations higher than 100, or 50 μg/L was calculated. 14.39% inpatients with S00-T98 reported blood Pb levels more than 100 μg/L, while 29.82% inpatients were over 50 μg/L. Inpatients with blood Pb levels over 100 μg/L accounted for merely 2.03% in C00-D48, and 2.92% in Z00-Z99. However, those whose blood Pb levels were higher than 50 μg/L accounted for 25.61% in C00-D48, and 28.33% in Z00-Z99 (Table 1).

**Table 1:** Diseases spectrum (ICD-10 Blocks) and prevalence rate of inpatients with blood Pb concentrations higher than 100 or 50 μg/L

Blocks	Diseases spectrum	Inpatients with Pb tests	≥100 μg/L	Percentage (%)	≥50 μg/L	Percentage (%)
A00-B99	Certain infectious and parasitic diseases	576	9	1.56%	77	13.37%
C00-D48	Neoplasms	246	5	2.03%	63	25.61%
D50-D89	Diseases of the blood and blood-forming organs and certain disorders involving the immune mechanism	252	3	1.20%	34	13.55%
E00-E90	Endocrine, nutritional and metabolic diseases	545	14	2.57%	93	17.06%
F00-F99	Mental and behavioural disorders	310	3	0.97%	29	9.35%
G00-G99	Diseases of the nervous system	1058	24	2.27%	168	15.88%
I00-I99	Diseases of the circulatory system	1955	25	1.28%	167	8.54%
J00-J99	Diseases of the respiratory system	3159	27	0.86%	543	17.22%
K00-K93	Diseases of the digestive system	1273	76	5.97%	313	24.59%
M00-M99	Diseases of the musculoskeletal system and connective tissue	349	8	2.31%	59	17.00%
N00-N99	Diseases of the genitourinary system	193	9	4.66%	39	20.21%
R00-R99	Symptoms, signs and abnormal clinical and laboratory findings, not elsewhere classified	249	18	7.23%	56	22.49%
S00-T98	Injury, poisoning and certain other consequences of external causes	285	41	14.39%	85	29.82%
Z00-Z99	Factors influencing health status and contact with health services	242	7	2.92%	68	28.33%

### Factors associated with blood Pb levels

The mean ± S.D. (range) age of patients in our study was 30 ± 19 (0–100) years old at the time of Pb measurement. Male or female patients accounted for 52.21% or 47.79% respectively. The median Pb levels of male or female patients were 35.31 μg/L or 29.70 μg/L, respectively. To identify the factors influencing Pb levels among inpatients, statistical differences of blood Pb concentrations were analyzed based on demographic factors. Except for blood type (ABO), significant difference was found among other six factors including gender, age, ethnic group, blood type (Rh), visit times, and treatment results. Male inpatients’ Pb levels was significantly higher than female (z-score = − 7.40, *p* < 0.01). Age group of 18 to 45 reported the highest Pb levels, compared with other groups. Pb levels of minority groups, 24.30 μg/L, were significantly higher than that of the Han inpatients, 20.21 μg/L (z-score = − 38.54, *p* < 0.01). Pb concentration of blood Rh negative-type was higher than positive-type (z-score = − 7.05, *p* < 0.01), but similar differences were not observed in the ABO blood types (χ^2^ = 6.54, *p* > 0.05). Those who were initially hospitalized reported blood Pb concentrations 24.00 μg/L, whereas those who were hospitalized more than twice reported a concentration of 19.11 μg/L. In terms of treatment results, Pb levels of cured, or improved inpatients were lower than invalid, untreated, or others (χ^2^ = 213.66, *p* < 0.01) (Table 2).

**Table 2:** Blood Pb levels at demographic factors, further in C34.901 or non-C34.901 among C00-D48 inpatients

Categorical Variables		No.	Percentage (%)	Pb levels	*p*-value	Statistics	No. of C34.901 (Pb levels)	No. of non-C34.901 (Pb levels)
Gender	Male	7645	57.23%	22.00	*p* < 0.01	−7.40^a^	15 (50.20)	101 (24.00)
	Female	5713	42.77%	20.00			15 (36.80)	105 (23.98)
Age	< 18	5042	37.83%	24.22	*p* < 0.01	596.34^b^	–	14 (24.50)
	18–45	2581	19.37%	25.00			1 (6.20)	52 (19.00)
	45–60	1839	13.80%	24.20			8 (53.03)	68 (26.92)
	> 60	3866	29.01%	16.00			21 (45.34)	78 (22.00)
Ethnic group	Han	12,762	95.17%	20.21	*p* < 0.01	−38.54^a^	30 (46.44)	205 (23.98)
	Minority	647	4.83%	24.30			–	7 (25.60)
Blood type (ABO)	A	1550	27.87%	20.00	*p* > 0.05	6.54^b^	3 (47.53)	48 (20.85)
	B	1696	30.49%	19.29			4 (25.11)	36 (25.00)
	O	1806	32.47%	20.11			2 (33.47)	50 (20.50)
	AB	510	9.17%	19.20			4 (32.75)	9 (37.14)
Blood type (Rh)	Negative	73	1.47%	43.47	*p* < 0.01	−7.05^a^	2 (72.45)	0
	Positive	4892	98.53%	19.33			13 (36.00)	131 (21.00)
Visit times	First	6770	50.49%	24.00	*p* < 0.01	−12.42^a^	13 (56.67)	115 (26.00)
	Not first	6638	49.51%	19.11			17 (30.00)	98 (20.55)
Treatment results	Cured	4890	36.56%	24.00	*p* < 0.01	213.66^b^	2 (43.91)	47 (25.00)
	Improved	7800	58.31%	19.39			8 (16.65)	127 (22.00)
	Invalid	41	0.31%	25.00			–	–
	Untreated	112	0.84%	26.00			1 (36.00)	9 (20.20)
	Died	55	0.41%	23.00			3 (15.11)	3 (32.00)
	Others	479	3.58%	33.80			16 (56.67)	27 (39.54)

C34.901, Malignant Neoplasm of Lung

C00-D48, Neoplasms

^a^ Z-score of two independent samples by Wilcoxon rank sum test

^b^ Chi-Squared values (χ^2^) of multiple independent samples by Kruskal-Wallis H test

After separating each demographic factor, we also measured Pb concentration between C34.901, and non-C34.901 within the block C00-D48 inpatients. C34.901 exceeded non-C34.901 in both male (50.20 μg/L vs 24.00 μg/L) and female patients (36.80 μg/L vs 23.98 μg/L) and only one C34.901 patient was reported younger than 45 years old. Interestingly, no significant differences were found between ABO blood types. The concentration of C34.901 patients with blood type A, O, AB were far higher than that of non-C34.901, while two groups with type B reported much close figures. In regard to the treatment results, cured C34.901 inpatients’ Pb levels, though with only two patients, markedly surpassed the improved, invalid, or untreated (Table 2).

## Discussion

In the present study, we investigated the blood Pb levels among the a massive number of patients with various diseases, and found that: (1) the average blood Pb concentrations of Chinese patients was 28.36 μg/L; (2) obvious geographical differences existed in blood Pb levels; (3) Han Chinese patients’ Pb levels were significantly lower than other minorities groups; (4) Pb exposure may be an important risk factor for malignant neoplasm of lung, considering the significantly higher Pb levels in lung cancer than other respiratory, and intrathoracic organs.

Compared with the blood Pb levels reported in previous research targeting occupational populations [4], children [3], or adults [16], Chinese patients as a whole, reported the lowest Pb levels, which to some extent represents conditions of residence throughout China. The findings may also be attributed to differences in the age structures of all the subjects. About 30% patients were over 60 years old, and their Pb concentration was the lowest compared with the rest three groups, 16.00 μg/L.

This study revealed the geographical differences of blood Pb levels in patients who visited hospitals, which could partially represent general population in China [1, 17]. Systematic review of published papers illustrated that, of 24 provinces, or regions, only 4 provinces (Hunan, Guangdong, Gansu, Jiangxi) showed a trend of increased mean blood Pb levels, and the prevalence of Pb poisoning [18]. However, two adjacent provinces, namely, Yunnan, and Guangxi Zhuang Autonomous Region, showed opposite trend in our survey, with Yunnan being the highest, and Guangxi the lowest. Similar results were observed not only in birthplace, but also in workplace, suggesting career as a factor related to Pb exposure. The fact that Yunnan has long been taking the first place in deposits of heavy metals may account for the Pb accumulation of patients born or worked in Yunnan [19]. Meanwhile, patients from Guangxi mainly dwelled in Guilin, a prefecture in the far south of China. This huge contrast of patients’ Pb levels between two regions may result from the heterogeneity of their living environment.

China is a multi-ethnic country. Han Chinese, as the majority, showed a significantly lower blood Pb levels than other minorities. The difference between Han group, and other groups has been pointed out in previous study, reporting a mean blood Pb levels of 55.70 μg/L, 53.00 μg/L in Uyger, and Han children, respectively [20]. This difference in Pb levels may be attributed to the varied living, and eating habits of ethnic groups. Remarkably, patients with Rh negative blood type seemed to accumulate more Pb than Rh positive. Blood serves as an important medium for Pb transport. More than 99% of blood Pb stays with red cells, while the rest is distributed in plasma [21]. Rh proteins form a core complex which is critical to the structure of the erythrocyte membrane [22], and might affect the sequestration, or excretion of Pb between red cells, and blood. Interestingly, Pb levels became lower when patients were hospitalized for a longer time. This may be attributed to medical interventions, professional health education, and guidance on behavior or nutrition from doctors during treatment.

In 2016, Pb exposure accounted for 540, 000 deaths and 13.9 million years of healthy life lost (disability adjusted life years, DALYs) worldwide due to long-term effects on health [23]. An environmentally attributable fraction model was used to estimate Pb-attributable total economic costs, $ 227.7 billion in Eastern Asia (China, Mongolia), represented by each 1-year cohort of children < 5 years of age [24]. DALYs are particularly useful for prioritizing public health interventions, meanwhile, total economic cost is used as a tool to assess medical burdens of a country or an area. Our study discovered that the average medical cost due to Pb poisoning was 6888 CNY, of which drug cost took the largest proportion, 4670 CNY. These findings suggested that the treatment of Pb poisoning, especially drug treatment, may be the major resource of medical burden for patients under Pb exposure, and average medical cost needs to be considered when assessing personal economic burden of residents, especially those in developing countries or areas.

Almost all the previous studies on the association between Pb exposure and lung cancer had Pb workers (among battery, or smelter workers) as their subjects. Besides, some studies reported confusing and even contradictory results on the associations. The estimated risk for lung cancer, as is commonly agreed, were elevated in Pb-only workers compared with other workers [25]. However, in two population-based case-control studies, investigators observed no increased risk of lung cancer with exposure to Pb compounds [26]. The present study further confirmed the association of Pb exposure with lung cancer among residents, in addition to occupational workers. However, the findings are limited because other possible factors affecting lung cancer risks were not considered.

This study reported a blood Pb concentration of 24.22 μg/L in patients under 18 years old. Teenagers, especially children, in their growth period, are sensitive to Pb exposure, which impairs their cognitive function, mental behavior, infant growth, or even influences future socioeconomic status [16, 27–29]. Guide issued by Ministry of Health to preventive measures against child-related high blood Pb levels and Pb poisoning set upper limit for children at 100 μg/L in China. However, Centers for Disease Control and Prevention (CDC) in America has set the upper limit for children at 50 μg/L in 2012. Therefore, we highly recommend that the critical value of blood Pb poisoning, especially for children, should be restricted to 50 μg/L in China.

Admittedly, this study has some limitations. Although we intended to investigate the current status of blood Pb levels all over China, there, to some extent, still existed flaws or loopholes in population selection. Those who have never tested their blood Pb concentration were not included in this study. The conclusion of the study representing whole population needs further verification. Furthermore, this study is not a case-control or cohort study in terms of methodology. Some conclusions, such as Pb as risk factor for lung cancer, need to be further verified in cohort study, or basic research. Finally, all data were limited in the HIS databases in the hospitals. Connections with patients’ other datasets outside hospitals will facilitate, and extend research of Pb exposure on human bodies.

## Conclusions

In summary, this study reported current status of blood Pb levels, its effects on diseases’ profile, and medical burden for patients who visited military hospitals, partially representing the whole Chinese populations. Despite the Chinese government’s efforts to control the environmental Pb level, Pb poisoning was still imposing the direct economic burdens on patients because of Pb exposure. From the perspectives of diseases occurrence, the association of Pb with lung cancer may open up new areas for Pb-induced toxicology, providing a basis for the government and public health departments to further control Pb levels. In summary, these findings reveal a novel insight into future toxicology research from the aspect of data mining techniques.

## Data Availability

The data that support the findings of this study are available from Directorate of Medical Services, Logistics Support Department of PLA, but restrictions apply to the availability of these data, which were used under license for the current study, and so are not publicly available. Data are however available from the authors upon reasonable request and with permission of Directorate of Medical Services, Logistics Support Department of PLA.

## Abbreviations

AAS: Atomic Absorption Spectroscopy
ADT: Admission-Discharge-Transfer
BBB: Blood-Brain Barrier
CDC: Centers for Disease Control and Prevention
CKD: Chronic Kidney Disease
DALYs: Disability Adjusted Life Years
EMR: Electronic Medical Record
HIS: Hospital Information System
ICD: International Statistical Classification of Diseases, and Related Health Problems
Pb: Lead
PRC: People’s Republic of China
SQL: Structured Query Language
